# Deciphering vancomycin resistance in *Enterococcus faecium*: gene distribution, sequence typing, and global phylogenetic analysis

**DOI:** 10.3389/fmicb.2025.1578903

**Published:** 2025-08-15

**Authors:** Ruyu Yan, Jun Ji, Han Shen, Xiaoli Cao

**Affiliations:** ^1^Department of Laboratory Medicine, Nanjing Drum Tower Hospital Clinical College of Nanjing University of Chinese Medicine, Nanjing, China; ^2^Department of Laboratory Medicine, Nanjing Drum Tower Hospital Clinical College of Nanjing University, Nanjing, China

**Keywords:** *Enterococcus faecium*, *van*, sequence types, phylogenetic tree, prevalence characteristics

## Abstract

**Objective:**

This study analyzes the global prevalence and distribution of vancomycin resistance genes (*van*) in *Enterococcus faecium* and examines the genetic relationship and epidemiological characteristics of strains carrying these genes.

**Method:**

A total of 3,256 *E. faecium* genome sequences were downloaded, and 2,235 high-quality genomes were retained after quality filtering. The blastn tool was used to screen these genomes for *van* genes, and sequence types (STs) were determined using pubMLST profiles.

**Result:**

Among the 2,235 genomes, 1,071 (47.9%) harbored *van* genes, with eight genotypes identified, including *vanA*, *vanB*, *vanD*, and *vanM*, accounting for 47.6%. There were 83 distinct STs among the strains carrying *van* genes, with ST17 being the most prevalent. Most strains carrying *van* genes were isolated from humans, primarily in the United States, and commonly from rectal swabs. In 2015, *vanA* was the most prevalent *van* gene, particularly in ST17 strains.

**Conclusion:**

This study highlights the widespread distribution of *van* genes and their significant presence in human populations and clinical settings, emphasizing the importance of monitoring and intervening in the spread of ST17 strains.

## 1 Introduction

*Enterococcus faecium* (*E. faecium*) is a common opportunistic pathogen in hospital settings, frequently responsible for infections of the blood, urinary tract, and abdome (Reynolds et al., 2021). The extensive use of vancomycin and other antibiotics has facilitated the emergence and proliferation of vancomycin-resistant *Enterococcus faecium* (VREfm) (Ji et al., 2024), a bacterium that poses significant challenges due to its limited treatment options, prolonged hospital stays, and high mortality rate (McHugh et al., 2024). Recognizing its critical impact, the World Health Organization included VREfm in its 2024 list of pathogens that urgently require new therapeutic approaches.

Resistance to vancomycin in VREfm is primarily mediated by vancomycin resistance (*van*) genes, which are typically located on plasmids (Li et al., 2022). The principal *van* genes in *Enterococcus*—*vanA*, *vanB*, *vanD*, and *vanM*—encode D-alanine:D-lactate ligase, conferring high-level resistance (Geraldes et al., 2022).

Epidemiological studies have identified distinct geographical and clonal patterns of these resistance genes. For example, Elstrøm et al. (2019) reported an incidence of 7.09 per 100,000 person-years for VRE infections/colonization in Norway (Eichel et al., 2023), while in Egypt, the pooled prevalence of VRE among clinical isolates as high as 26%, with *E. faecium* reaching 32.5% (Azzam et al., 2023). A meta-analysis in Nigeria found that 63.1% of *E. faecium* strains exhibited resistance to vancomycin (Orababa et al., 2021). Moreover, different regional studies highlight the prevalence of specific sequence types (STs) associated with these resistance genes: in Brazil, the *vanA* gene predominantly occurs in ST21 strains (Farias et al., 2023); in Poland, nearly all carriers of the *vanA* gene (97.6%) are associated with the Clonal Complex CC2 and CC87 (Wardal et al., 2022); in Algeria, the *vanA* gene mainly appears in ST80 and ST789, part of CC17 (Benamrouche et al., 2021); in Germany, the *vanB* gene is most frequent, particularly in ST117 (Nürnberger et al., 2021); and in China, a study from a tertiary hospital found the *vanA* gene most prevalent in ST78, ST192, and ST570 (Zhou et al., 2020). However, data on the global prevalence and characteristics of *van* genes among *E. faecium* remain limited.

With the increased use of vancomycin, the number of strains carrying *van* genes has risen and rapidly evolved. The advancement of whole-genome sequencing (WGS) technology has also led to a significant increase in sequenced bacterial genomes, with *van* resistance genes being increasingly reported, potentially establishing *E. faecium* carrying *van* genes as a common pathogen (Karaman et al., 2020). However, global distribution data on *E. faecium* carrying *van* genes remain limited. In this study, we first explored the distribution of *van* genes in *E. faecium* isolates based on global databases. For strains carrying *van* genes, we further investigated STs, evolutionary relationships, as well as their regional and temporal distribution.

## 2 Materials and methods

### 2.1 Genome sequence download and pre-processing

*E. faecium* genome sequences (GenBank format) were systematically retrieved in batch format using Aspera^1^ as of the cutoff December 7, 2023. The GenBank files for all 3,256 *E. faecium* genomes were downloaded using Perl script. All 3,256 strains were annotated using Prodigal, chosen for its robustness in gene prediction, to ensure consistency across the dataset. Quality filtering was performed using CheckM v1.1.3 and Quest 5.0.2 software (Li et al., 2023; Joddha et al., 2023). We set specific parameters to ensure the selection of high-quality genomes: integrity greater than 90%, contamination rate less than 5%, a maximum of 500 contigs per genome, and an N50 of at least 40 kb (Wattanasombat and Tongjai, 2024). These stringent criteria ensured that only the most reliable and complete genomes were included for further analysis. After rigorous filtering, 2,235 high-quality genomes were obtained. These genomes met all specified quality standards and were deemed suitable for in-depth genetic and epidemiological analysis.

### 2.2 Identification of vancomycin resistance genes

We compiled a structured resistance gene database using *van* resistance gene sequences sourced from the NCBI Pathogen Resistance Gene Database. The nucleotide coding sequences for all genes in the 2,235 high-quality genomes, as annotated by Prodigal, were compared against our structured resistance gene database using blastn. This comparison was aimed at obtaining a detailed distribution of *van*-positive genes across all analyzed genomes. To ensure specificity and relevance of the matching results, we set stringent parameters for the blastn analysis: *E*-value = 1 × 10^−5^, identity ≥90%, coverage ≥90%, match length ≥30.

### 2.3 Sequence typing of *van*-carrying *Enterococcus faecium* strains

For the sequence typing of 1,071 *van*-carrying *E. faecium* strains, a self-designed tool, ST-tool was utilized. This tool was developed specifically to handle the unique demands of our study, ensuring accurate and efficient sequence typing based on established genetic markers. Seven housekeeping gene sequence files for *E. faecium* from the pubMLST website were acquired. These files represent the core of our sequence typing analysis, as they contain critical genetic markers used for identifying STs among different *E. faecium* isolates. Using blastn, we compared the housekeeping gene sequences from genomes carrying *van* genes against the seven housekeeping gene sequence files obtained. The criteria for this comparison were stringent, requiring 100% identity and 100% coverage to ensure that only exact matches were considered. This high threshold was set to accurately assign sequence types without ambiguity. The results from the blastn comparisons were then cross-referenced with the profile file from pubMLST to determine the STs of each strain. By matching gene sequences to known profiles, we could assign an ST to each *van*-carrying *E. faecium* strain, facilitating further epidemiological and resistance mechanism analysis.

### 2.4 Phylogenetic tree construction

Two phylogenetic trees were constructed, one from 915 genomes containing the *vanA* gene and another from 130 *E. faecium* genomes containing the *vanB* gene. Initially, genomic nucleotide sequence files were downloaded in batch from the NCBI genome database using the specified genome assembly numbers. The *E. faecium* genomes were annotated using Prokka version 1.14.6 (Zhao et al., 2022), and the resulting GFF files served as input for Roary version 3.13.0 (Sultana et al., 2022), which performed a metagenomic analysis to yield core gene multiple sequence alignment files. From these, single nucleotide polymorphism (SNP) sequence files were extracted using SNP-sites v2.5.1 (Coll et al., 2022). Subsequently, the optimal nucleotide substitution model, GTR + G, was determined using jModelTest 2 based on the SNP sequence files (Jacob Machado et al., 2021). A maximum likelihood phylogenetic tree was then constructed for each gene group using RAxML-NG v. 1.2.1 software (Togkousidis et al., 2023), employing the GTR + G model with 1,000 bootstrapping samples. The resulting phylogenetic trees were imported into iTOL Version 6.5.8 software (Letunic and Bork, 2024), where branches with bootstrap values less than 50 were removed. The trees were exported as SVG files, which were then enhanced visually using Adobe Illustrator. Throughout this process, 487 core gene multiple sequence alignment files were acquired from the 915 genomes containing *vanA*, and 1,156 core gene multiple sequence alignment files were obtained from the 130 genomes containing *vanB*.

### 2.5 Metadata extraction and integration

We extracted meta information from the GenBank files of 2,235 genomes using custom Perl scripts. This metadata included critical epidemiological and clinical details such as the isolation time, country of origin, host, and sample source of each strain. The extracted metadata was then integrated with STs obtained from the sequence typing analysis. To facilitate this integration, both datasets were compiled into a single table, allowing for a comprehensive dataset that combines genetic and epidemiological data.

## 3 Results

### 3.1 A high prevalence of the *van* gene in global *Enterococcus faecium* populations

Among the 2,235 strains of *E. faecium* analyzed, 1,071 (47.9%) were found to carry the *van* gene. In all *E. faecium*, *vanA* was the most prevalent, identified in 915 strains (40.9%), followed by *vanB* in 130 strains (5.8%). Additionally, *vanD* was detected in 15 strains (0.7%), *vanM* in 12 strains (0.5%), and *vanP* in four strains (0.2%). The *vanN* gene was identified in two strains (0.1%).

### 3.2 Multiple sequence types serve as hosts for *van* genes

**Figure 1:**
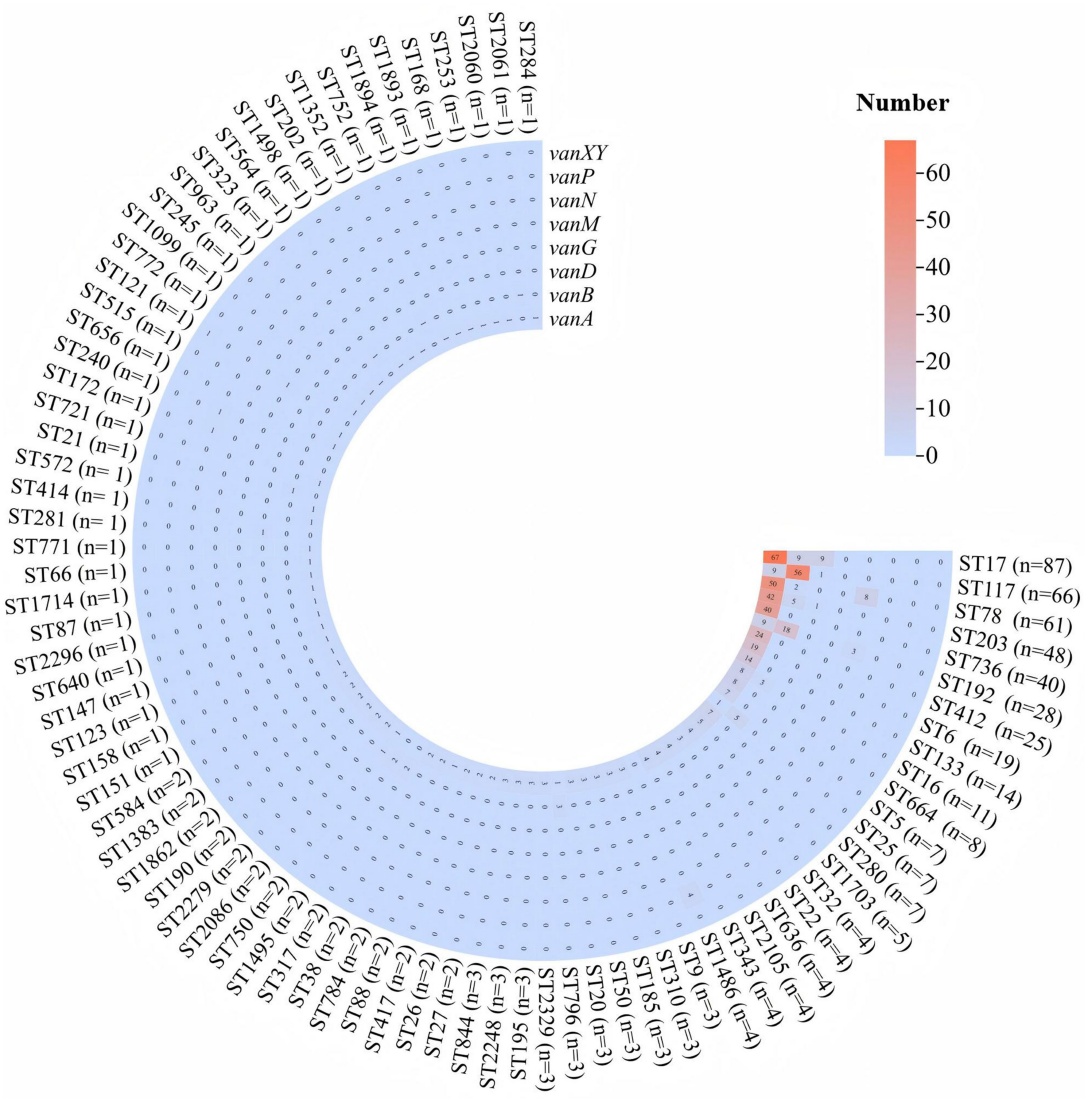
Distribution of vancomycin resistance genes among the predominant sequence types of *Enterococcus faecium*. The heatmap displays the presence of six vancomycin resistance genes: *vanA*, *vanB*, *vanD*, *vanG*, *vanN*, *vanP*, and *vanXY*. Each row represents a specific ST, with the number of isolates indicated in parentheses. The color gradient, from blue to red, indicates the number of isolates carrying each resistance gene, with higher numbers shown in red and lower numbers in blue.

In total, 83 different STs were identified among the 1,071 *E. faecium* strains carrying *van* gene. Among them, ST17 was the most common, found in 87 (8.1%) strains, followed by ST117 (6.1%) in 66 strains, ST78 in 61 (5.7%) strains, ST203 in 48 (4.5%) strains, and ST736 in 40 (3.7%) strains, the distribution of the major *van* genotypes such as *vanA*, *vanB*, *vanD*, and *vanM*, along with STs, is shown in Figure 1.

## 4 Genetic relationship

### 4.1 Phylogenetic tree for strains carrying *vanA* gene

**Figure 2:**
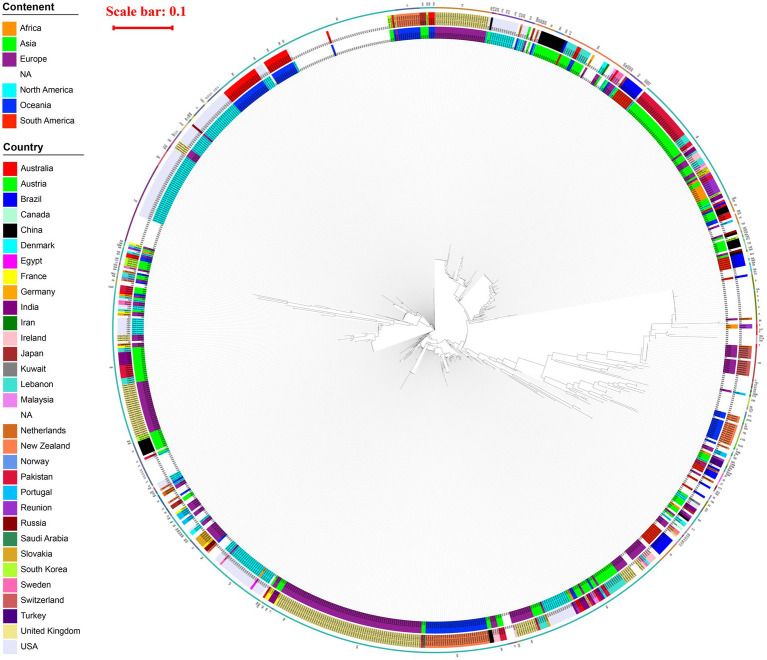
Phylogenetic tree of 915 *vanA*-carrying *Enterococcus* isolates circular maximum likelihood phylogenetic tree of 915 *vanA*-carrying *Enterococcus* isolates. The innermost ring indicates the continent of origin, the middle ring shows the country, and the outermost ring represents the MLST sequence type (ST) of each isolate. A red scale bar (0.1) indicates genetic distance. The tree reveals extensive geographic and genetic diversity, with multiple clades corresponding to both regional clusters and widespread international lineages. STs such as ST17, ST78, and ST203 are frequently observed and often associated with specific geographic regions.

A total of 915 strains carrying *vanA* gene were analyzed through whole-genome phylogenetic reconstruction, revealing distinct genetic clusters alongside geographic and ST information, as shown in Figure 2. The innermost ring of the circular phylogenetic tree indicated that the majority of isolates originated from Europe, followed by Asia and North America, with fewer contributions from Africa, Oceania, and South America. Some isolates lacked geographic metadata and were labeled as NA. Country-level data, represented in the middle ring, showed high proportions of isolates from the United Kingdom, Germany, the USA, France, the Netherlands, and Australia, with additional contributions from countries such as China, Japan, India, and Saudi Arabia. These isolates formed both geographically concentrated clusters—suggesting local outbreaks—and mixed-country branches, indicating potential international transmission. The outermost ring showed a wide diversity of STs, with frequent representation of ST17 and ST78 across multiple regions. These STs often formed dense clusters, implying both local clonal expansion and the global spread of dominant *vanA*-carrying lineages. Overall, the phylogenetic structure highlighted a combination of localized hospital-associated outbreaks and widespread dissemination of high-risk clones across continents.

### 4.2 Phylogenetic tree for strains carrying *vanB* gene

**Figure 3:**
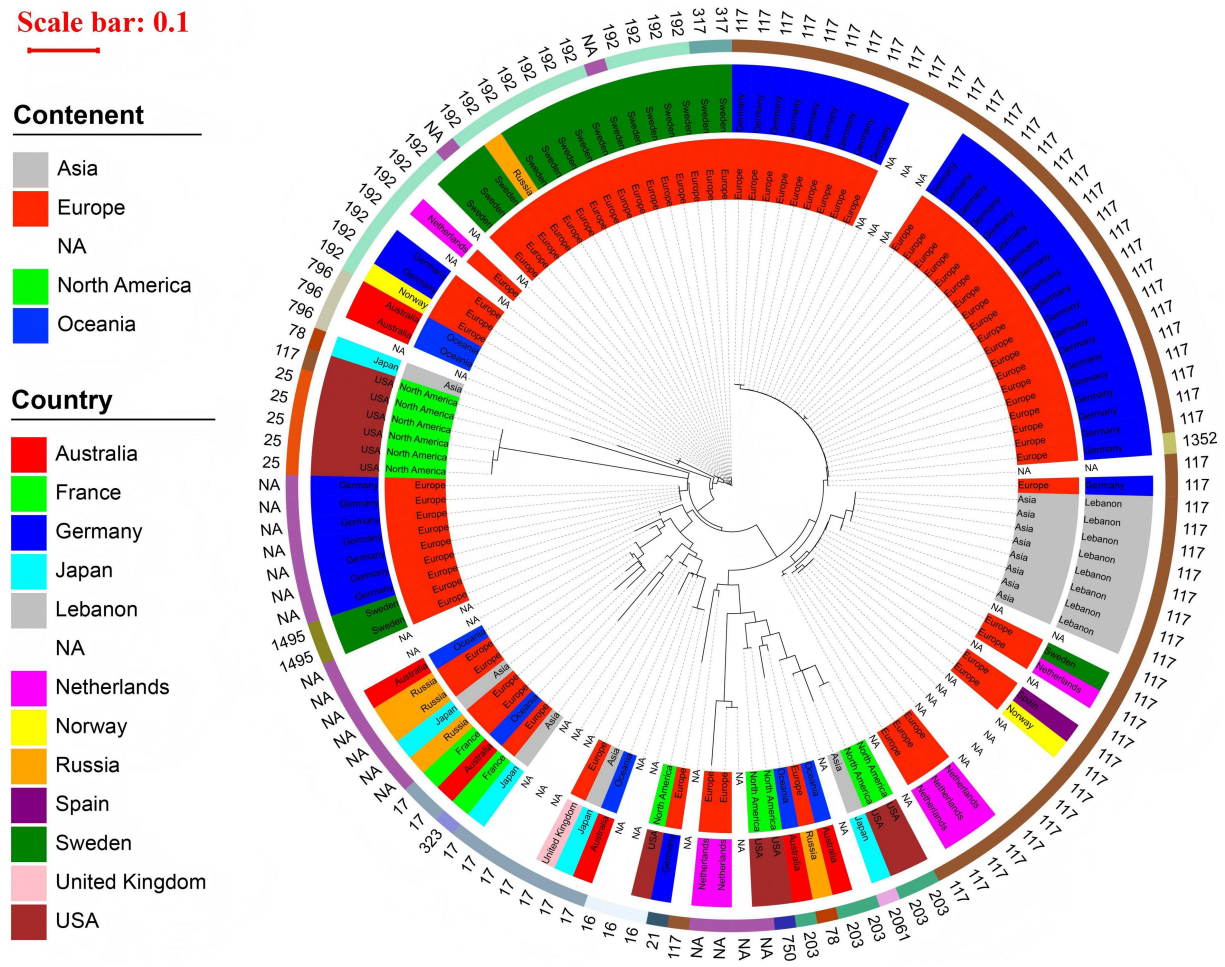
Phylogenetic tree of 130 *vanB*-carrying *Enterococcus* isolates circular maximum likelihood phylogenetic tree of 130 *vanB*-carrying *Enterococcus* isolates. The innermost ring represents the continent, the middle ring indicates the country, and the outermost ring displays the MLST sequence type (ST) of each isolate. A red scale bar (0.1) shows the genetic distance. The tree is dominated by a large ST117 clade composed primarily of isolates from Germany and other European countries. Additional STs—including ST203, ST17, and ST192—form smaller, geographically associated clusters. The tree highlights both localized clonal expansion and international dissemination of *vanB*-carrying strains.

In the phylogenetic tree of 130 strains carrying *vanB* gene, we found that the innermost ring of the circular phylogenetic tree showed that most isolates originated from Europe, with additional contributions from Asia, North America, and Oceania; several were labeled as NA due to missing metadata. At the country level, Germany accounted for the largest number of isolates, forming a prominent clonal cluster, while other countries such as the United Kingdom, USA, France, Australia, Japan, and Lebanon were also represented. The outermost ring indicated a strong dominance of ST117, which formed a large, closely related clade, primarily composed of isolates from Germany and other European countries. Other STs, including ST17, ST203, and ST192, were present in smaller numbers and often clustered geographically. These findings suggest a major clonal expansion of ST117 within Europe—particularly in Germany—alongside evidence of both localized outbreaks and international dissemination of *vanB*-carrying lineages, as shown in Figure 3.

## 5 Human was the predominant host for *Enterococcus faecium* carrying *van* gene

Among the 1,071 *E. faecium* strains carrying *van* gene analyzed, human sources accounted for the highest proportion, comprising 61.3% (*n* = 657) of all isolates. The most common human-derived samples included stool (*n* = 288), blood (*n* = 118), and urine (*n* = 33). Animal and dairy-related samples constituted 2.5% (*n* = 27) of the isolates, with the *Gallus gallus* domesticus being the most prevalent (*n* = 14), followed by pigs (*n* = 6), dogs (*n* = 2), and cats (*n* = 2), as detailed in Table 1.

**Table 1:** The hosts and sample types of *van*-positive *Enterococcus faecium* isolates with known origin.

Hosts (*n*)	Sample types (*n*)
Animal and dairy-related samples (27)	Gallus gallus domesticus (14), pigs (6), dogs (2), cats (2), piggery environmental water (1), camel milk (1), Dahi fermented milk product (1)
Humans-related samples (657)	Stool (288), other samples of human (148), blood (118), urine (33), infection (9), routine screen (8), laboratory strain (5), wound (4), peritoneal fluid (3), clinical laboratory (3), clinical (3), superficial wound (3), hospitalized patient (2), sanies (2), bile (2), airways (1), ascites (1), pleura effusion (1), fecal (1), organic material (1), cerebrospinal fluid (1), jejunal aspirate (1), hospital surface (1), gastrointestinal tract (1), abdominal wound (1), drain fluid (1), pus (1), foot wound (1), catheter (1), hospital effluent (1), tissue (1), pleural fluid (1), knee swab (1), screening (1), drain liquid UVI (1), ulcer (1), wound secretion (1), muscle (1), sputum (1), EQA test strain (1), hepatic abscess (1)
Environment-related samples (73)	Bedside rail in hospital intensive care unit (12), hospital other source samples (12), alcohol foam dispenser in hospital intensive care unit (10), nursing call button in hospital intensive care unit (10), bedside light switch in hospital intensive care unit (7), wastewater (7), washroom sink in hospital intensive care unit (5), stream surface water (5), sewage (4), river surface water (1)
Other (314)	Blank (280), patient sample (34)

## 6 Distributional characteristics of strains carrying *van* genes

### 6.1 Geographical distribution of strains carrying *van* genes

**Figure 4:**
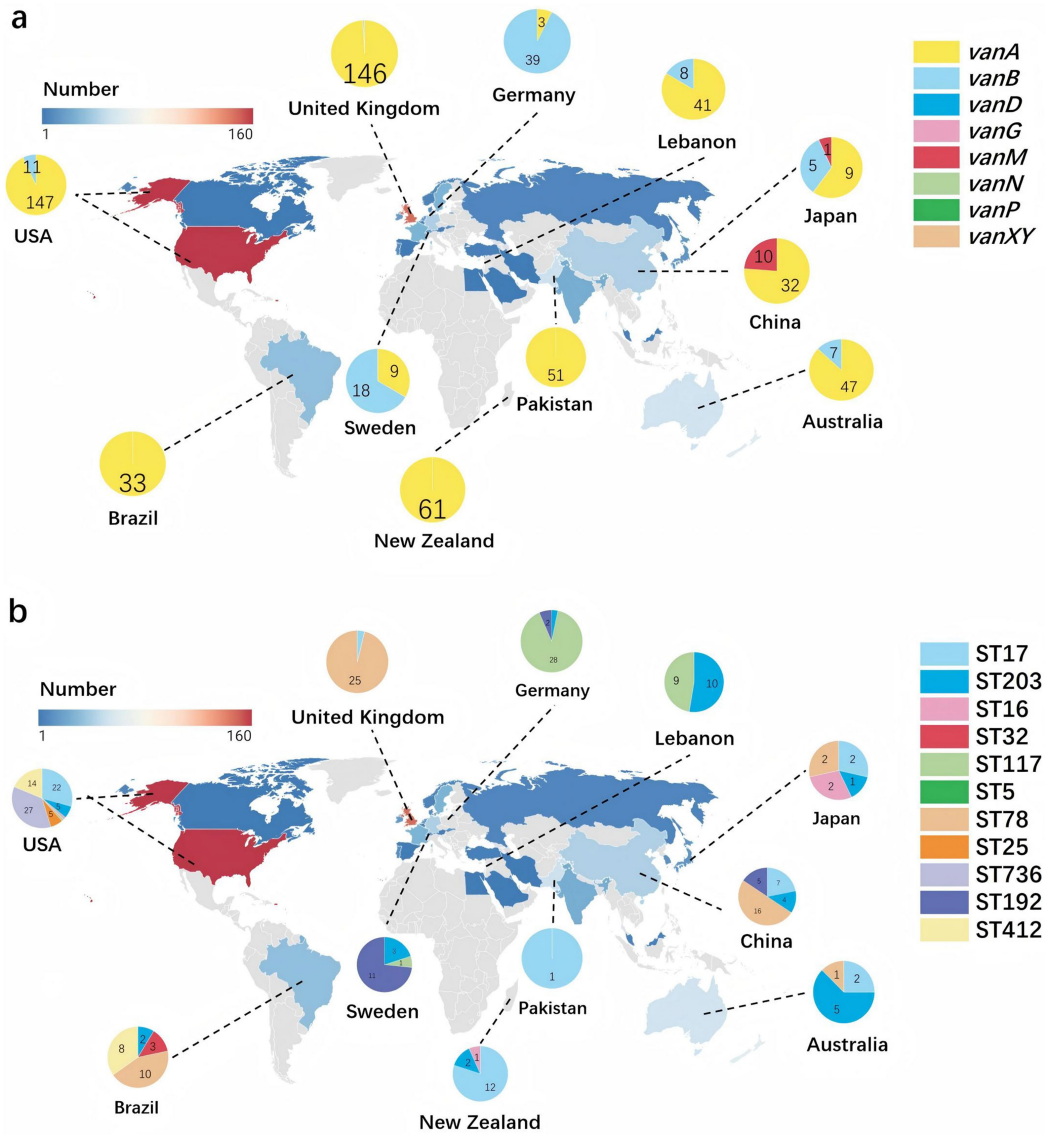
Global distribution of *Enterococcus faecium* dominant sequence types and vancomycin resistance genes. The color gradient indicates gene frequency, with red representing higher counts and blue representing lower counts. The geographic distribution of all strains included in the study is covered. **(a)** Highlights the distribution of eight different *van* gene subtypes, focusing on the 11 countries with the highest *van* gene frequencies. Each segment of the pie chart is color-coded according to the vancomycin resistance gene, with the legend on the right indicating the corresponding colors for *vanA*, *vanB*, *vanD*, *vanG*, *vanM*, *vanN*, *vanP*, and *vanXY*. **(b)** Uses pie charts to show the distribution of the 10 most prevalent STs, displaying only the 11 countries with the highest *van* gene frequencies on the map. Each segment of the pie chart is color-coded according to the ST, with the legend on the right indicating the corresponding colors for ST17, ST203, ST16, ST32, ST117, ST5, ST78, ST25, ST736, ST192, and ST412.

From the 2,235 genomes of *E. faecium*, a total of 1,071 *E. faecium* carrying *van* gene were obtained, with their geographical distribution detailed in (Supplementary Table S1). Notably, the United States recorded the highest number of isolates at 160, followed by the United Kingdom with 148 isolates, and New Zealand with 62 isolates. In the United States, the most common STs were ST736 (*n* = 27), ST17 (*n* = 22), and ST412 (*n* = 14), the predominant *van* is *vanA* (91.9%), followed by *vanB* (6.9%). In the United Kingdom, ST78 was the most prevalent (*n* = 25), followed by ST280 (*n* = 4) and ST17 (*n* = 1), with *vanA* being the predominant *van* gene (98.6%). Meanwhile, in New Zealand, ST17 was the most frequently found (*n* = 12), followed by ST2105 (*n* = 4) and ST195 (*n* = 3), with *vanA* predominating as the most common *van* gene (98.4%) as shown in Figure 4.

### 6.2 Annual distribution of strains carrying *van* genes

**Figure 5:**
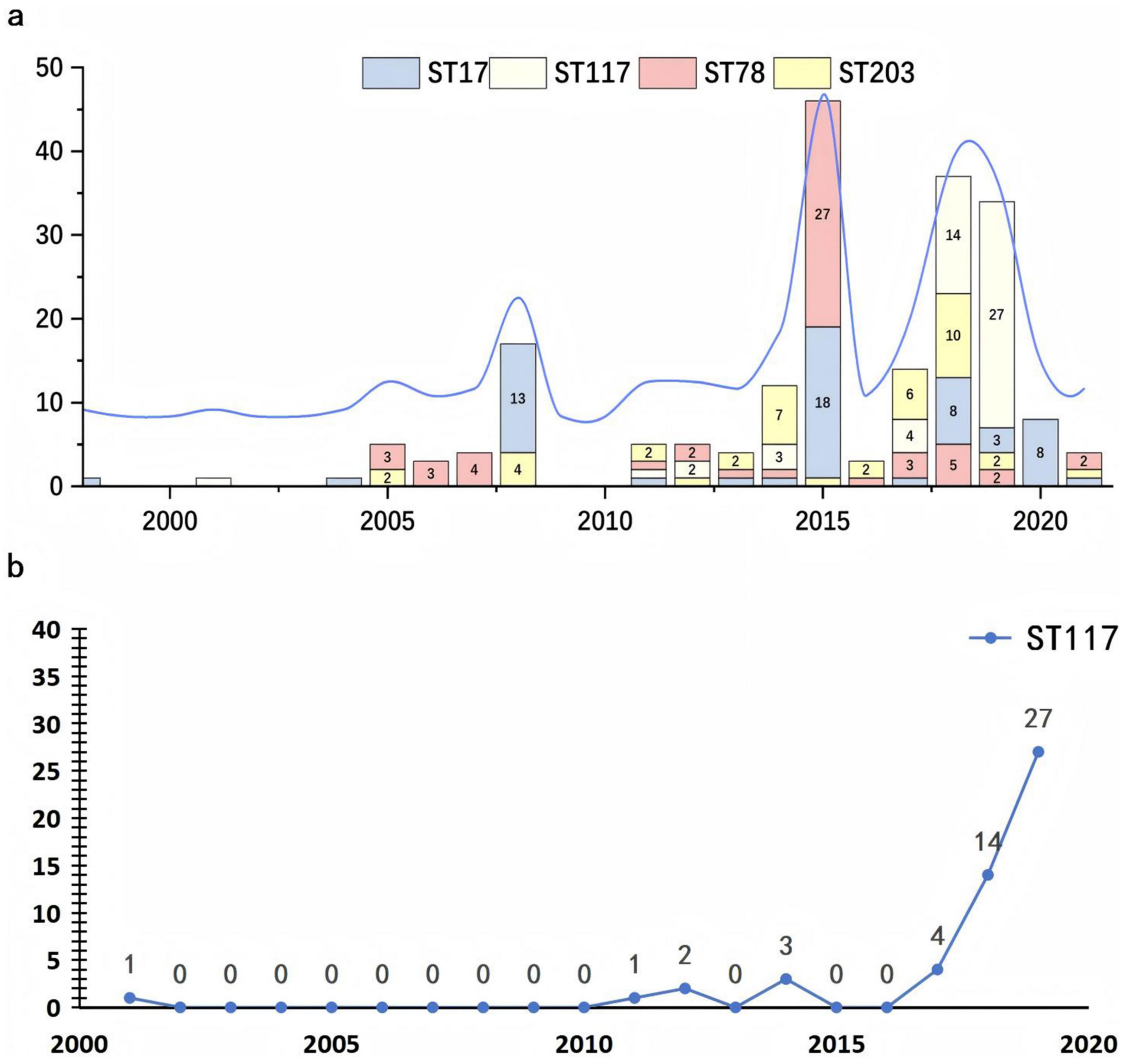
Temporal distribution of major sequence types of *Enterococcus faecium* from 2000 to 2020. **(a)** The stacked bar chart illustrating the annual number of isolates for each sequence type (ST), with distinct colors representing ST17 (blue), ST117 (yellow), ST78 (red), and ST203 (gray). An overlaid blue line indicates the total number of isolates per year. **(b)** The trend chart depicting the yearly distribution of ST117 *E. faecium* carrying *van* genes.

The initial isolations of *E. faecium* strains ST17 and ST117 occurred in 1998 and 2001, respectively, while strains ST203 and ST78 were first identified in 2005. Starting in 2008, the dissemination rate of ST17 began to increase significantly, exhibiting fluctuations but maintaining a consistently high presence in subsequent years. By 2015, the ST78 clone had gradually emerged as the dominant clone. However, a notable increase in the dissemination rate of ST117 was observed between 2018 and 2019, leading to its establishment as the most prevalent clone during that period, as shown in Figure 5.

## 7 Discussion

Vancomycin remains a critical antimicrobial agent against multidrug-resistant *E. faecium* infections. However, resistance to this vital drug presents substantial challenges in clinical settings. This study provides valuable insights into the epidemiological distribution and characteristics of vancomycin resistance, which are crucial for developing clinical treatment strategies and preventive measures. Understanding these patterns can help healthcare providers better manage and prevent vancomycin-resistant infections.

Our analysis identified a 47.9% prevalence rate of *van* genes in *E. faecium*, with *vanA* being the most frequent variant (40.9%), based on global data collected from 1995 to 2022. When comparing with other studies, however, it is critical to consider differences in sample origin, geographic scope, detection method, and study objectives. For instance, our *vanA* prevalence is lower than the 44.7% observed in municipal wastewater samples from Germany (Valenza et al., 2024), but higher than rates from clinical isolates in Toronto (10.2%), Australia (13.7%) (Coombs et al., 2024), and Japan (0.9%) (Kohler et al., 2018). In Beijing hospitals, *vanA* and *vanM* prevalence were reported at 24.4 and 4.7% respectively (Yan et al., 2023), while all VREfm strains in a Turkish study carried *vanA* (Erdem et al., 2020). These variations highlight the importance of interpreting prevalence differences with caution, as biases may arise from inconsistent sampling contexts (e.g., wastewater vs. clinical), database submission trends, and genomic screening criteria. Additional *van* genes detected in our dataset include *vanB* (5.8%), *vanD* (0.7%), *vanM* (0.5%), *vanP* (0.2%), and *vanN* (0.1%). These frequencies differ from localized hospital-based reports—for example, *vanB* prevalence of 36.8–47.6% in a German hospital (Jochim-Vukosavic et al., 2024), *vanM* at 16.7% in a tertiary Chinese hospital (Zhou et al., 2020), and *vanD* at 27.8% in the Netherlands (Flipse et al., 2019). Again, such discrepancies underscore the limitations of direct cross-study comparison without careful consideration of sample origin, sequencing depth, and local epidemiology. Importantly, the presence of multiple *van* gene variants—each conferring resistance through different mechanisms such as inducible ligase systems (VanA/VanB) or intrinsic modifications (VanC)—indicates a high potential for heterogeneity in clinical outcomes and detection sensitivity (Karukappadath et al., 2023).

Beyond human isolates, our metadata analysis identified a subset of strains derived from non-human hosts, including poultry, pigs, dogs, and environmental sources (e.g., wastewater, surface water). Although these occurrences were infrequent, the detection of *vanA*- and *vanB*-carrying strains from animal and environmental sources suggests potential reservoirs for resistance genes. These findings align with previous studies identifying zoonotic and ecological reservoirs for VREfm (Leinweber et al., 2018), and emphasize the need for a One Health approach to resistance surveillance that integrates human, veterinary, and environmental domains.

Our multilocus sequence typing (MLST) analysis revealed that ST17, ST117, and ST78 were the most prevalent STs among *vanA*-carrying isolates. Notably, ST117 has gained increasing attention due to its growing presence in recent years. In our phylogenetic tree, ST117 formed several well-supported clades—especially in isolates from Europe, North America, and Oceania—suggesting clonal expansion. However, we caution against overinterpreting this trend: the apparent increase in ST117 may reflect temporal bias in genome submissions rather than true epidemiological replacement of ST17. To mitigate this, we normalized ST frequencies by total genome counts per year, and observed a consistent proportional rise in ST117 from 2010 onwards. Still, we interpret this as a potential trend rather than a definitive lineage shift, as longitudinal genomic data and *in vitro* fitness validation are lacking.

The biological success of ST117 may be multifactorial. Prior studies have identified several adaptive traits in ST117 strains, including plasmid-mediated metabolic flexibility (e.g., PTS sugar transporters), virulence factors (e.g., pili, secretion systems), and enhanced colonization abilities under antibiotic pressure (Stege et al., 2024). ST117 is frequently associated with Inc18 and RepA_N plasmids that stably harbor *vanA*, potentially facilitating its expansion in hospital environments (Al Rubaye et al., 2023). Nevertheless, in the absence of experimental evidence on growth dynamics or transmission efficiency, we can only hypothesize that ST117’s rise is driven by both fitness advantage and selection pressure in healthcare settings.

These high-risk clones formed well-supported clusters that were either geographically restricted—suggesting localized outbreaks and sustained transmission within specific healthcare systems—or dispersed across countries and continents, indicative of clonal spread via inter-hospital or international transfer of patients (Permana et al., 2023). Particularly, the wide geographic reach of ST17 supports the role as pandemic clones capable of persisting and disseminating under strong antibiotic selection pressure (Willems et al., 2011; van Hal et al., 2021). The application of Roary allowed us to identify core-genome-based phylogenetic structure (Page et al., 2015), while RAxML-NG enabled robust tree construction, revealing both deeply rooted lineages and evidence of recent diversification (Kozlov et al., 2019). Some inconsistencies between phylogeny and geography, especially in mixed-country branches, may reflect recombination events or horizontal acquisition of resistance genes, including *vanA*, which is commonly located on transmissible plasmids and transposons (e.g., Tn1546) (Gouliouris et al., 2018). The potential for plasmid-mediated spread of *vanA* among different STs within shared environments underscores the complexity of resistance dissemination beyond clonal inheritance (Gorrie et al., 2019).

In parallel, the phylogenetic reconstruction of 130 *vanB*-carrying isolates demonstrated a marked clonal dominance of ST117, especially among German and other European strains. This suggests not only a successful regional expansion of a healthcare-adapted lineage, but also potential plasmid stabilization of *vanB* within this genetic background (Lisotto et al., 2021; Falgenhauer et al., 2019). Other STs—such as ST203, ST17, and ST192—formed smaller, geographically constrained clades, suggesting independent introduction events and localized outbreaks (Lehmkuhl et al., 2024; Hughes et al., 2019; Lam et al., 2013). The ST117-dominant clade exhibited very short branch lengths, indicative of recent expansion likely driven by clonal spread within hospitals or healthcare networks under antimicrobial pressure (Xanthopoulou et al., 2020). The detection of genetically similar *vanB*-carrying isolates in Asia, North America, and Oceania suggests that international dissemination is also occurring, possibly via mobile genetic elements and intercontinental transfer of colonized patients (Wei et al., 2024; Santona et al., 2023; Bender et al., 2022). However, due to the relatively small number of *vanB* isolates, further analysis—especially of the accessory genome and plasmid content—would be necessary to confirm the role of mobile elements and recombination in their spread (Mills et al., 2025).

Together, these findings underscore the importance of integrating phylogenomic, epidemiological, and mobile genetic element data to fully understand the transmission dynamics of *vanA* and *vanB* resistance genes. The observed patterns of clade formation and geographic clustering, supported by core-genome phylogenies, highlight both long-standing epidemic lineages and ongoing regional transmission events, reinforcing the need for real-time genomic surveillance and infection control efforts, particularly targeting high-risk clones such as ST117 (Hourigan et al., 2024).

Our study has several limitations. First, the representation of *E. faecium* strains in the NCBI database may not be comprehensive, introducing potential selection bias and limiting the generalizability of our findings. Second, the lack of susceptibility data restricts our ability to correlate genotypes with phenotypes accurately, hindering a full understanding of how genetic variations affect clinical outcomes. Further research should focus on including data from underrepresented regions and exploring the interactions between genetic diversity and resistance mechanisms.

## 8 Conclusion

Using whole-genome analysis of 2,235 strains, we identified major epidemic clones such as ST17, ST78, and ST117, with ST117 showing recent expansion, especially in *vanB*-carrying populations. The observed clonal structure, combined with evidence of recombination and potential plasmid-mediated gene transfer, underscores the complex transmission dynamics of vancomycin resistance. Our findings emphasize the dual role of local hospital-associated outbreaks and global dissemination in shaping the current landscape of VREfm. These insights reinforce the urgent need for real-time genomic surveillance, coordinated international monitoring, and targeted infection control measures—particularly in healthcare settings—to curb the spread of high-risk resistant clones.

## Data Availability

The datasets presented in this study can be found in online repositories. The names of the repository/repositories and accession number(s) can be found in the article/Supplementary material.

## Footnotes

^1^ https://www.ncbi.nlm.nih.gov
