# Supplementary material for: Deciphering vancomycin resistance in Enterococcus faecium: gene distribution, sequence typing, and global phylogenetic analysis
Source: Front Microbiol. 2025 Aug 15;16:1578903. doi: 10.3389/fmicb.2025.1578903 (PMC12394535; doi:10.3389/fmicb.2025.1578903)
Supplement: Supplementary file 1 [file Table_1.docx]

**Table 1**

The hosts and sample types of *van*-positive *Enterococcus faecium* isolates with known origin.

| Hosts (n) | Sample types (n) |
| --- | --- |
| Animal and dairy-related samples(27) | Gallus gallus domesticus (14), pigs (6), dogs (2), cats (2), piggery environmental water (1), camel milk (1), Dahi fermented milk product (1) |
| Humans-related samples (657) | stool (288), other samples of human (148), blood (118), Urine (33), Infection (9), routine screen (8), laboratory strain (5), wound (4), Peritoneal fluid (3), Clinical laboratory (3), clinical (3), superficial wound (3), Hospitalized patient (2), sanies (2), bile (2), airways (1), ascites (1), pleura effusion (1), fecal (1), organic material (1), cerebrospinal fluid (1), jejunal aspirate (1), hospital Surface (1), gastrointestinal tract (1), abdominal wound (1), drain fluid (1), pus (1), foot wound (1), catheter (1), hospital effluent (1), tissue (1), pleural fluid (1), knee Swab (1), screening (1), drain liquid UVI (1), ulcer (1), wound secretion (1), muscle (1), sputum (1), EQA test strain (1), hepatic abscess (1) |
| Environment-related samples (73) | bedside rail in hospital intensive care unit (12), hospital other source samples (12), alcohol foam dispenser in hospital intensive care unit (10), nursing call button in hospital intensive care unit (10), bedside light switch in hospital intensive care unit (7), wastewater (7), washroom sink in hospital intensive care unit (5), stream surface water (5), sewage (4), river surface water (1) |
| Other (314) | blank (280), patient sample (34) |
